# Ochratoxin A induces ER stress and apoptosis in mesangial cells via a NADPH oxidase-derived reactive oxygen species-mediated calpain activation pathway

**DOI:** 10.18632/oncotarget.14270

**Published:** 2016-12-27

**Authors:** Meei-Ling Sheu, Chin-Chang Shen, Yuan-Siao Chen, Chih-Kang Chiang

**Affiliations:** ^1^ Department of Medical Research, Taichung Veterans General Hospital, Taichung, Taiwan; ^2^ Institute of Biomedical Sciences, National Chung Hsing University, Taichung, Taiwan; ^3^ Rong Hsing Research Center for Translational Medicine, National Chung Hsing University, Taichung, Taiwan; ^4^ Chemical Engineering Division, Institute of Nuclear Energy Research, Atomic Energy Council, Longtan District, Taoyuan, Taiwan; ^5^ Institute of Toxicology, College of Medicine, National Taiwan University, Taipei, Taiwan; ^6^ Department of Integrated Diagnostics & Therapeutics, National Taiwan University Hospital, College of Medicine, National Taiwan University, Taipei, Taiwan

**Keywords:** ochratoxin A, mesangial cells, NADPH oxidase, ER stress, apoptosis

## Abstract

Ochratoxin A (OTA) contaminated food increases reactive oxygen species (ROS) production in glomerulus and causes glomerulopathy. The molecular mechanisms still remain uncertain. In this study, we used mouse and rat glomerular mesangial cells and delineate the signaling pathway behind the OTA-triggered cell apoptosis. OTA dose-dependently induced expression of ER stress markers including phospho-PERK, phospho-eIF2α, GRP78, GRP94, and CHOP. Apoptosis events including cleavage of caspase-12, caspase-7, and PARP are also observed. OTA activated oxidative stress and increased NADPH oxidase activity. NADPH oxidase inhibitor, apocynin, significantly attenuated OTA-induced cell apoptosis. Moreover, OTA markedly increased the calpain activity which significantly inhibited by apocynin. Transfection of calpain-siRNA effectively inhibited the OTA-increased ER stress-related protein expression. These findings suggest that OTA activated NADPH oxidase and calpain, induced ER stress and ROS production, and caused glomerular mesangial cells apoptosis which leads to glomerulopathy.

## INTRODUCTION

Ochratoxin A (OTA), a widely-spread mycotoxin produced by fungi, is a contaminant in the food chain worldwide. OTA is a secondary fungal metabolite and is known to be nephrotoxic. It has been suspected to be a major etiological substance for Balkan endemic nephropathy (BEN) and urinary tract cancer [1]. The pathological characterizations of BEN include a progressive atrophy and sclerosis in the kidney [2]. The glomerular and vascular lesions can also be observed in BEN, which include ischemic, microcystic, and obsolescent glomeruli, occasional thrombotic microangiopathy-like lesions, and focal segmental sclerosis-like lesions [2]. The renal lesions in porcine nephropathy, which OTA is a major causal determinant, are also characterized by the degeneration of the proximal tubules, interstitial fibrosis and hyalinization of the glomeruli [3]. The regional thickening and degeneration of the glomerular basement membrane has also been found in broiler chicks fed OTA [4]. Recently, Ciarcia et al. has shown that OTA treatment presented hypertension and reduction of glomerular filtration rate in rats [5]. However, the molecular mechanisms involved in the OTA-induced glomerulopathy still remain uncertain.

OTA induced oxidative stress and apoptosis in renal proximal tubular cells [6], monkey and human kidney epithelial cells [7], and kidney *in vivo* [8]. Endoplasmic reticulum (ER) stress-induced renal cell apoptosis caused some kidney diseases [9]. Mycotoxin patulin induced human intestinal and kidney cell cytotoxicity through an oxidative stress-related induction of ER stress and mitochondrial apoptosis pathway [10]. The roles of oxidative stress and ER stress in OTA-induced glomerular mesangial cell cytotoxicity still remain unclear. In this study, we investigated the cytotoxic effect and molecular mechanism of OTA on glomerular mesangial cells. The involvements of oxidative stress, ER stress, and apoptosis in OTA-triggered mouse and rat mesangial cell cytotoxicity were tested.

## RESULTS

### OTA decreased cell viability and induced apoptosis and markers for ER stress and apoptosis in mesangial cells

**Figure 1:**
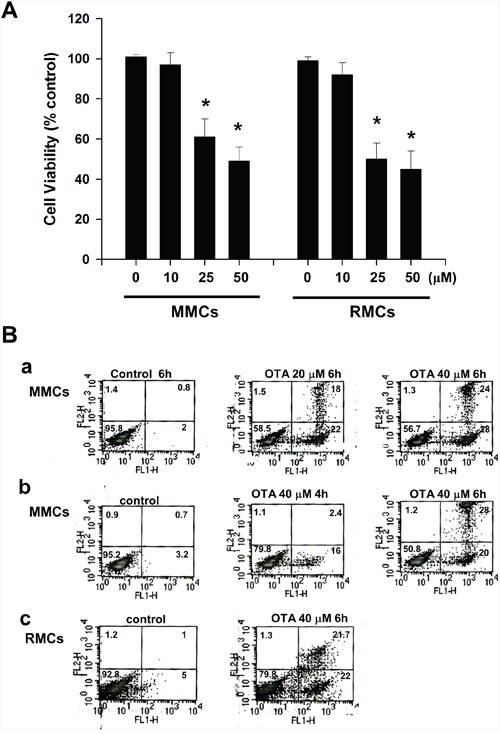
Effects of ochratoxin A (OTA) on cell viability and apoptosis in mouse mesangial cells (MMCs) and rat mesangial cells (RMCs) In **A**. cells were treated with OTA (10-50 μM) for 24 h. Cells were subjected to the MTS assay for determination of the cell viability. Data are presented as means ± SEM from three to five independent experiments performed in duplicate. * p < 0.05 as compared with control. In **B**. cells were analyzed by annexin V/PI staining for apoptosis. (a), MMCs treated with OTA (20 and 40 μM) for 6 h. (b), MMCs treated with OTA (40 μM) for 4 h and 6 h. (c), RMCs treated with OTA (40 μM) for 6 h. The percentage of cells found in each quadrant of the dot plot is depicted. Results shown are representative of at least three independent experiments.

We first tested the effects of OTA on cell viability and apoptosis in mesangial cells (MMCs and RMCs). OTA (10-50 μM) dose-dependently decreased MMCs and RMCs cell viability after 24 h exposure (Figure 1A). Annexin-V/PI staining also showed that OTA effectively induced apoptosis in MMCs and RMCs (Figure 1B).

**Figure 2:**
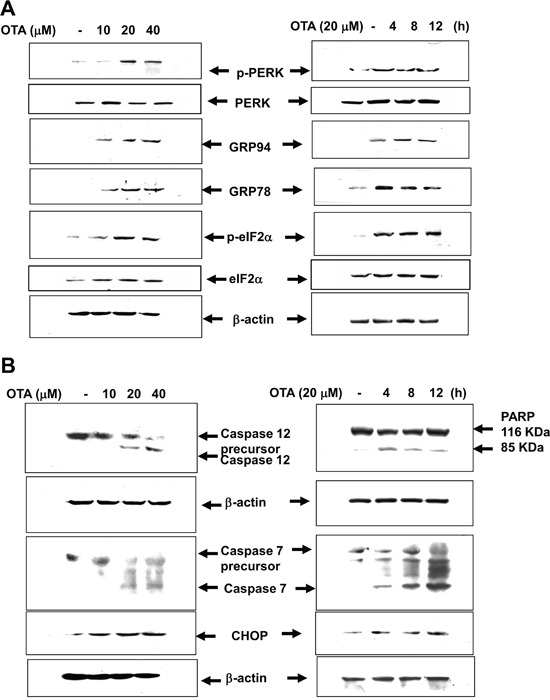
Induction of markers of ER stress and proapoptosis in OTA-treated mesangial cells MMCs were treated with OTA (10-40 μM) for 4-8 h. ER stress markers (**A**. phospho-PERK, PERK, phospho-eIF2α, eIF2α, GRP78, and GRP94) and ER stress-related proapoptotic markers (**B**. CHOP and cleavages of caspase-12, caspase-7, and PARP) were determined by Western blotting. Results shown are representative of at least three independent experiments.

Excessive ER stress triggers cellular apoptosis. We further to examine whether induced ER stress was essential for OTA-induced apoptosis. The markers for ER stress and apoptosis, such as phospho-PKR-like ER kinase (p-PERK), phospho-eukaryotic initiation factor-2α (p-eIF2α), GRP78, GRP94, CHOP, and cleavages of caspase-12, caspase-7, and PARP (poly-ADP-ribose polymerase) were investigated. As shown in Figure 2A and 2B, MMCs were treated with OTA (10-40 μM) for 4-12 h. OTA markedly induced the expressions of ER stress and apoptosis makers in MMCs in a dose- and time dependent manner.

### OTA stimulated ROS production and NADPH oxidase activity in mesangial cells

**Figure 3:**
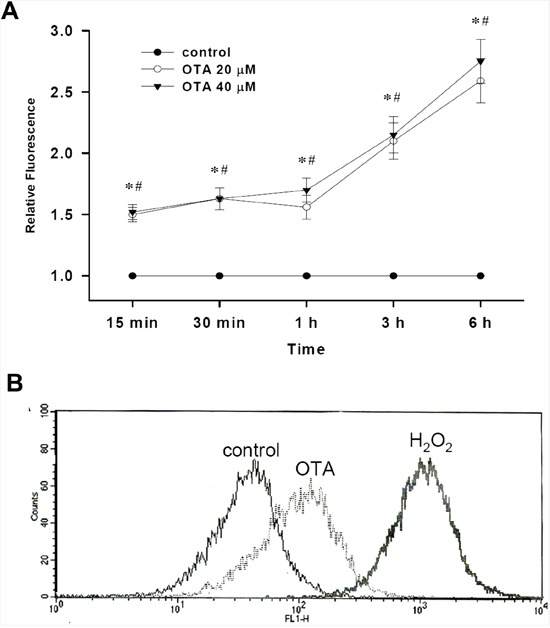
Effect of OTA on ROS generation in mesangial cells Intracellular ROS was determined by fluorescence of DCFH-DA as described under Material and Methods. In **A**. MMCs were treated with OTA for various time intervals as indicated, and then the ROS generation was detected. Data are presented as means ± SEM from three to five experiments performed in duplicates. * p < 0.05 as OTA 20 μM group compared with control. # p < 0.05 as OTA 40 μM group compared with control. In **B**. RMCs were treated with 20 μM OTA for 1 h. The relative fluorescence intensity was determined by flow cytometry. H2O2 (100 μM) was as a positive control. Results shown are representative of at least three independent experiments.

Next, we determined whether OTA stimulates ROS production in mesangial cells. As shown in Figure 3A, OTA (20 and 40 μM) induced ROS generation in MMCs as early as 15 min and gradually increased up to 6 h. Similarly, treatment of OTA (20 μM) in RMCs for 1 h also increased the ROS production (Figure 3B).

**Figure 4:**
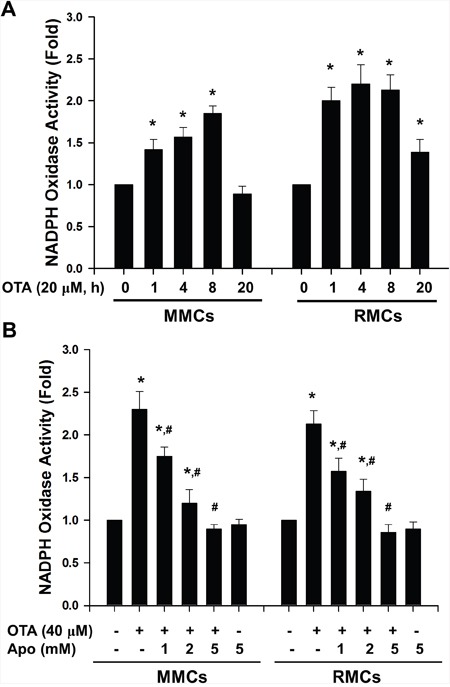
OTA induced NADPH oxidase activity in mesangial cells MMCs and RMCs were treated with 20 μM OTA for 1-20 h **A**. or were treated with 40 μM OTA for 4 h in the presence or absence of apocynin (1-5 mM). **B**. NADPH oxidase activity was determined by chemiluminescence assay as described under Material and Methods. All data are presented as means ± SEM from three independent experiments performed in duplicate. * p < 0.05 as compared with control. # p < 0.05 as compared with OTA alone.

NADPH oxidase is the major sources of ROS in variety of cells. We further determined whether OTA stimulated ROS generation via the NADPH oxidase-dependent pathway. As shown in Figure 4A, OTA (20 μM) significantly increased the NADPH oxidase activity in MMCs and RMCs in a time-dependent manner. NADPH oxidase inhibitor apocynin significantly and dose-dependently inhibited the increased NADPH oxidase activity in MMCs and RMCs treated with OTA (Figure 4B).

### Apocynin attenuated OTA-induced cell death and apoptosis

**Figure 5:**
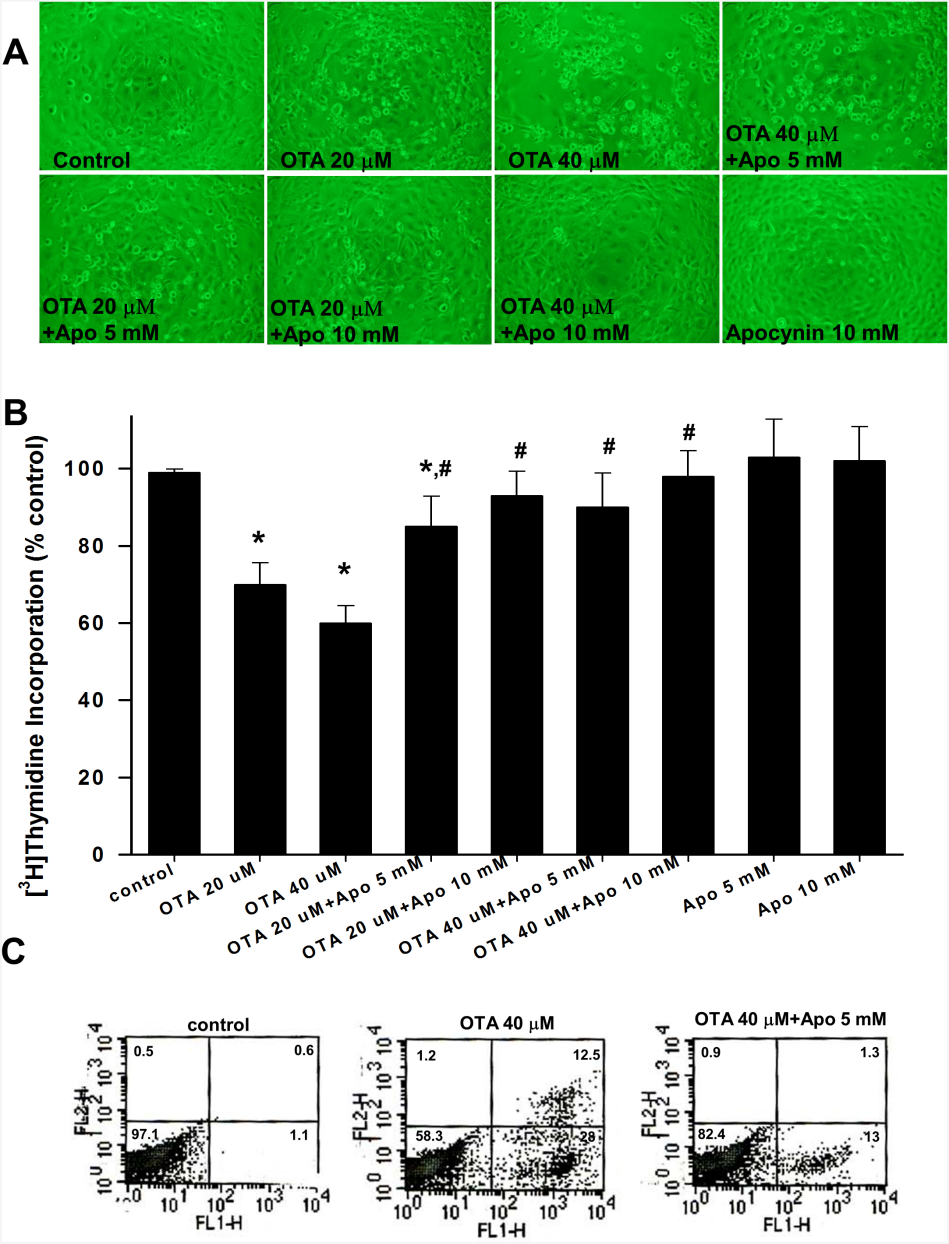
Effects of apocynin on cell growth and apoptosis in mesangial cells In **A** and **B**. MMCs were treated with OTA (20 μM and 40 μM) for 24 h in the presence or absence of apocynin (5 and 10 mM). The cell morphology (A) and cell proliferation (B) were observed. For determination of cell proliferation, cells were subjected to the [^3^H]thymidine incorporation as described under Material and Methods. Data are presented as means ± SEM from three to four independent experiments performed in duplicate. * p < 0.05 as compared with control. # p < 0.05 as compared with OTA alone. In **C**. MMCs were treated with OTA (40 μM) for 6 h in the presence or absence of apocynin (5 mM). Cell apoptosis was determined by annexin V/PI staining. The percentage of cells found in each quadrant of the dot plot is depicted. Results shown are representative of at least three independent experiments.

We next investigated whether NADPH oxidase-dependent ROS production was involved in the OTA-induced mesangial cell cytotoxicity. To address this issue, we determined the effects of apocynin on cell growth and apoptosis in OTA-treated mesangial cells. Bright field image observation revealed that apocynin (5 and 10 mM) protected cell growth from OTA (20 and 40 μM)-induced cytotoxicity in MMCs (Figure 5A). Treatment of OTA (20 and 40 μM) resulted in a suppression of cell proliferation determined by [^3^H]thymidine incorporation in MMCs (Figure 5B). Pretreatment ofapocynin (5 and 10 mM) could significantly inhibit the OTA-decreased cell proliferation in MMCs (Figure 5B). Apocynin (5 and 10 mM) had no effect on the basal proliferation of MMCs. Moreover, apocynin (5 mM) could also inhibit the OTA (40 μM)-induced cell apoptosis in MMCs (Figure 5C).

### OTA induced calpain activity in mesangial cells, which could be inhibited by apocynin

**Figure 6:**
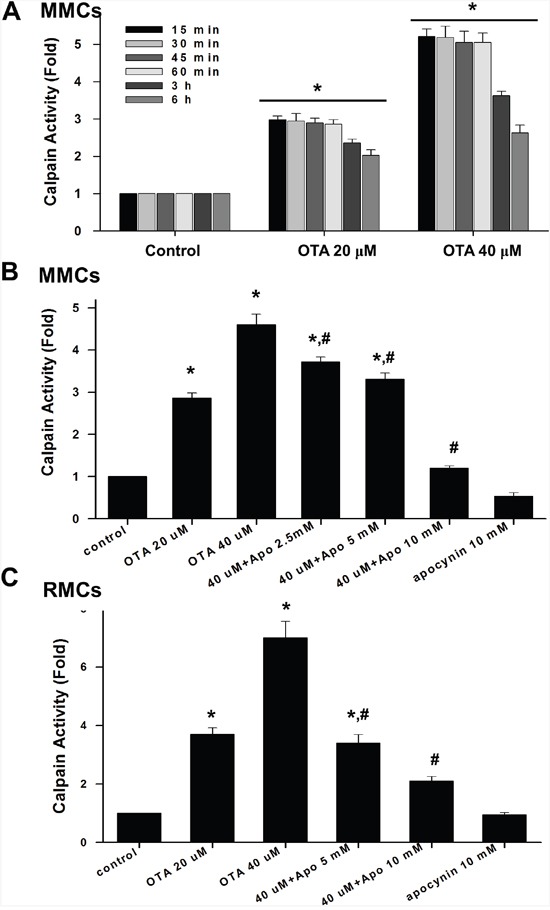
OTA induced calpain activity in mesangial cell **A**. MMCs were treated with OTA (20 and 40 μM) for 15 min-6 h. **B**. MMCs were treated with OTA (20 and 40 μM) for 1 h in the presence or absence of apocynin (5 and 10 mM). **C**. RMCs were treated with OTA (20 and 40 μM) for 1 h in the presence or absence of apocynin (5 and 10 mM). Calpain activity was measured by calpain substrate Suc-Leu-Leu-Val-Tyr-AMC as described under Material and Methods. All data are presented as means ± SEM from three to five independent experiments performed in duplicate. * p < 0.05 as compared with control. # p < 0.05 as compared with OTA alone.

We next investigated the effect of OTA on calpain activity in mesangial cells. As shown in Figure 6, OTA (20 and 40 μM) significantly increased the calpain activity in MMCs (Figure 6A and 6B) and RMCs (Figure 6C). Apocynin (2.5-10 mM) significantly reduced the OTA-increased calpain activity in mesangial cells (Figure 6B and 6C).

**Figure 7:**
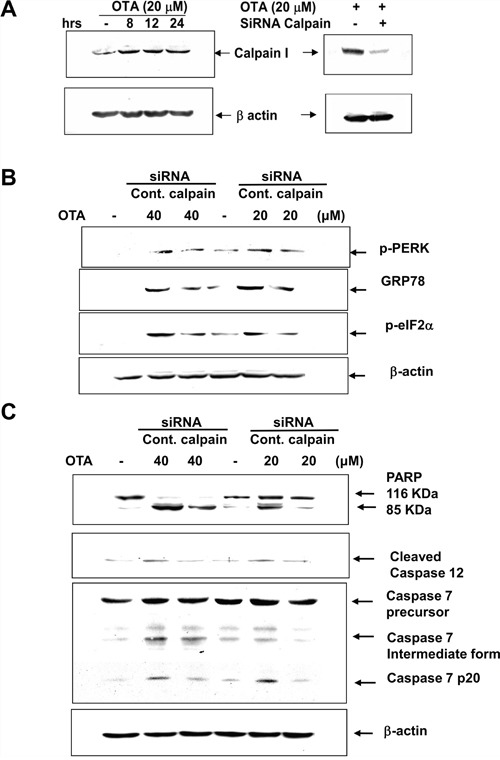
Transfection of calpain siRNA inhibited OTA-induced ER Stress and proapoptotic markers MMCs were transfected with control- or calpain-siRNA for 36 h. MMCs were treated with OTA (20 and 40 μM) for 8 h. The proteins (**A**. calpain I; **B**. phospho-PERK, phospho-eIF2α, and GRP78; **C**. cleavages of PARP, caspase-12, and caspase-7) were determined by Western blotting. Results shown are representative of at least three independent experiments.

The activity of calpain is as a key event in a variety of disorders, which connect to ER stress. Therefore, we next examined the role of calpain in OTA-induced markers for ER stress and apoptosis. The siRNA-mediated gene-silencing to knockdown calpain in mesangial cells was used. As shown in Figure 7, transfection of calpain siRNA led to a significant abatement in OTA-activated ER stress markers (p-PERK, p-eIF2α and GRP78) (Figure 7A) and proapoptotic molecules (cleavages of caspase-12, caspase-7, and PARP) (Figure 7B). These results demonstrated that NADPH oxidase was involved in the OTA-activated calpain activity-induced ER stress and apoptosis in mesangial cells.

## DISCUSSION

OTA is produced by fungi from improperly stored foods. It has been analyzed that the average weekly intake of OTA varies from 130 to 6489 ng in inhabitants from an area with high BEN incidence in Bulgaria [11–12]. The Joint FAO/WHO Expert Committee on Food Additives (JECFA) has established a provisional tolerable weekly intake (PTWI) for OTA of 100 ng/kg bw/week [13]. A prevalence of blood OTA levels exceeding 2 ng/ml was found in BEN-affected families in Bulgaria [14]. A high level of serum OTA (1.8 μg/ml) has been found in one of the Croatian samples [15]. The concentrations of OTA in serum and kidney tissue of pigs from endemic areas of Bulgaria were 27-249 ng/ml (66.8-616 nM) and 1.32±1.25 μg/kg, respectively [16–17]. It has been reported that blood levels of OTA are 0.7-7.8 ng/ml in the general population and 12-55 ng/ml in the patients with chronic renal failure in Tunisia [18]. Experimental feeding of OTA (0.5 mg/kg), which total plasma concentration of OTA was 12.2±0.44 μM, has been shown to cause a reduction of glomerular filtration rate (GFR) and of para-aminohippuric acid clearance in pigs [19]. Treatment of OTA (0.5 mg/kg, i.p.) in rats could also lead to a reduction of GFR [20]. The cellular and molecular mechanism of OTA-induced glomerulopathy needs to be clarified. In the present study, we found that OTA was capable of inducing cytotoxicity in glomerular mesangial cells. An oxidative stress-stimulated ER stress-related cell apoptosis pathway was involved in the OTA-triggered mesangial cytotoxicity.

OTA has been suggested to act on different sites along the nephron according to the evidence of pathophysiological investigations [21]. In the kidney, ROS can be generated in various cells [22]. The IC50 for cell viability of OTA in the proximal tubule cells and LLC-PK1 cells under normal medium was approximately 50 μM; the increased ROS contributed to the OTA-induced proximal tubular cytotoxicity [6]. OTA (5-40 μM) treatment has been found to activate signal-regulating kinase 1 (ASK1), increase ROS generation, and suppress cell viability in human embryonic kidney cells HEK293 [23]. It has been shown that OTA at a concentration of 1 μM significantly decreases the protein content in human mesangial cells; the cellular mechanism remains unknown [24]. OTA metabolism through cytochrome P450 requires high level of nicotinamide adenine dinucleotide phosphate (NADPH) which leads to activated pentose phosphate pathway (PPP) [25]. Excess of glucose entry is diverted through the PPP which possibly provided additional substrate for the enzyme NADPH oxidase [26]. Moreover, in the kidney, NADPH oxidases are known to be a distinct cellular localization [22]. It has been shown that the predominant source of superoxide production in the rabbit renal cortex is NADPH oxidase [27].

In the present study, we found that OTA at the concentrations of 10-50 μM was capable of inducing cytotoxicity and cell apoptosis in mouse and rat mesangial cells. OTA could also induce ROS generation and NADPH oxidase activity. NADPH oxidase inhibitor apocynin significantly inhibited the OTA-induced mesangial cytotoxicity and apoptosis. Several studies have demonstrated that apocynin has therapeutic effect on multiple animal disease model including chemically-induced colitis in mice [28], testicular ischemia-reperfusion injury in rats [29], bleomycin-induced lung fibrosis in rats [30], Cyclosporine A-Induced hypertension and nephrotoxicity in rats [31], established alcoholic steatohepatitis rat model [32], and contrast-induced nephropathy in the diabetic rats [33]. Therefore, these findings indicate that the concentrations of OTA used in this cellular toxicological study are reasonable and effectively induce mesangial cell cytotoxicity via a NADPH oxidase-derived ROS-mediated pathway.

ER stress can be induced in various renal diseases. ER stress-induced renal cell apoptosis is known to be one of the major causes of certain renal diseases [9]. Mycotoxin patulin (5 to 25 μM) has been shown to induce cytotoxicity in human colon carcinoma HCT116 cells and embryonic kidney HEK293 cells through a ROS-induced ER stress and induction of mitochondrial apoptotic signaling pathway [10]. Mycotoxin zearalenone (10-100 μM) could also induce cytotoxicity in RAW 264.7 macrophages through an ER stress pathway [34]. Moreover, calpain, an intracellular Ca^2+^-dependent cysteine protease, has been indicated to be associated with renal cell death induced by renal toxins [35–36]. Calpain activation has also been suggested to contribute to the ER stress-associated renal cell death following renal toxicants exposure [37]. Previous studies showed that increased ROS induces calpain activation in retinal photoreceptor cells which trigger the cell apoptosis [38]. ROS can target ER-based calcium channels leading to increased release of calcium into cytosol [39]. Since NADPH oxidase is the major ROS production enzyme inside cells therefore we hypothesized that NADPH oxidase activates calpain through ROS-induced calcium accumulation in cell cytosol. In the present study, we found that OTA (20-40 μM) effectively activated calpain activity in mesangial cells, which could be inhibited by apocynin. Transfection of calpain siRNA significantly inhibited the OTA-induced ER stress and apoptosis-related signaling molecules. These results suggest that calpain activation is involved in the induction of ER stress and apoptosis in OTA-treated mesangial cells.

In conclusion, this study demonstrated for the first time that OTA is capable of inducing oxidative stress, ER stress, apoptosis, and cell death in glomerular mesangial cells. NADPH oxidase and calpain activation are elevated in OTA-induced ER stress and apoptosis/cell death in mesangial cells, which may be involved in the pathogenesis of OTA-induced glomerulopathy.

## MATERIALS AND METHODS

### Cell culture

Cell line murine glomerular mesangial MES-13 cells (MMCs) and primary rat mesangial cells (RMCs) were used in this study. Dulbecco's modified Eagle's medium (DMEM) supplemented with 5% fetal bovine serum (FBS), 100 U/ml penicillin, 100 μg/ml streptomycin, and 14 mM HEPES was used to culture cells unless otherwise stated. Cells were routinely passaged by trypsinization after they reached 80% confluence using 10-cm culture dishes and incubating them at 37°C in a humidified chamber with a 5% CO_2_/95% air mixture. RMCs were obtained by culturing glomeruli isolated from kidneys of 100- to 150-g male Sprague-Dawley rats by conventional sieving methods as described previously [30]. Cells were cultured in DMEM containing 20% fetal bovine serum (FBS), 100 U/ml penicillin, 100 μg/ml streptomycin, 44 mM NaHCO_3_, and 14 mM HEPES.

### Cell viability

Viability was measured using the Cell Titer 96™ AQueous cell viability assay kit (Promega, Madison, WI, USA). This assay is based on the cellular conversion of the colorimetric reagent MTS (3,4-(5-dimethylthiazol-2-yl)-5-(3-carboxymethoxy-phenyl)-2-(4-sulfophenyl)-2 H-tetrazolium salt) in the presence of electron-coupling reagent phenazine methosulfate into soluble formazan by dehydrogenase, which was only found in metabolically active condition in living cells. Formazan formation was measured on the basis of increased absorbance at 490 nm.

### [^3^H]Thymidine incorporation

DNA synthesis was measured by incorporation of [^3^H]thymidine into cellular DNA. Cells were seeded in 96-well microtiterplates in a density of 5 × 10^4^ cells/ml in medium containing 5% FBS. After attachment of the cells overnight, [^3^H]thymidine was added to the medium (1 μCi/ml), and then cells were incubated for an additional 18 h. At the end of the labeling period, the cells were washed twice with PBS, and harvested onto glass filters with an automated 96-well glass fiber harvester (PerkinElmer, Waltham, MA, USA). The radioactivity retained on the filter was measured by a scintillation β-counter (PerkinElmer).

### Annexin-V FITC and PI double staining

The annexin V/propidium iodide (PI) (Takara Bio, Mountain View, CA, USA) was used to quantify numbers of apoptotic cells. Cells were washed twice with PBS and stained with annexin V and PI for 20 min at room temperature. The level of apoptosis was determined by measuring the fluorescence of the cells by flow cytometry analysis. (Becton Dickinson, San Jose, CA). Data acquisition and analysis were performed by the CellQuest program (Becton Dickinson).

### Measurement of NADPH oxidase activity

Superoxide production was measured in total cell homogenates by using lucigenin-derived chemiluminescence as described previously [31]. Briefly, 50 μg of protein was diluted in 500 μl of 50 mM phosphate buffer containing 1 mM EGTA and 150 mM sucrose. Dark-adapted lucigenin was added to the sample, and chemiluminescence measurement was immediately started. Chemiluminescence (in arbitrary units) was measured at 15 s intervals for 1 min in a Tropix TR717 luminometer. NADPH (100 μM), a necessary substrate for NADPH oxidase, was added to optimize the detection NADPH oxidase-related superoxide anion. The protein levels were measured by the BCA Kit.

### Detection of intracellular reactive oxygen species (ROS)

Intracellular ROS generation was detected by flow cytometry using peroxide-sensitive 2′, 7′-dichlorofluorescein diacetate (DCFH-DA) fluorescent probe (Thermo Fisher Scientific, Waltham, MA, USA). In brief, cells (5 × 10^5^) were incubated with 50 μM DCFH-DA. DCFH-DA was converted by intracellular esterases to DCFH. In the presence of the proper oxidant, DCFH was oxidized into the highly fluorescent 2′, 7′-dichlorofluorescein (DCF). After incubation, cells were resuspended in ice-cold PBS and placed on ice in darkness for flow cytometry analysis.

### Calpain activity assay

Cells were cultured in 24-well plates. Cells were loaded with 40 μM Suc-Leu-Leu-Val-Tyr-AMC (a calpain protease substrate) and treated with OTA for indicated timing at 37°C in a humidified 5% CO_2_ incubator. Quantitation of 7-amino-4-methylcoumarin (AMC) fluorescence can be used to measure enzyme activity. Proteolysis of the fluorescent probe was monitored using a fluorescent plate reading system (BioAssay Systems, Hayward, CA, USA) with filter settings of 360 ± 20 nm for excitation and 460 ± 20 nm for emission.

### Calpain siRNA transfection assay

Calpain 1 siRNA (sc-29886; Santa Cruz Biotechnology, Dallas, Texas, USA) was used for transient transfection of mesangial cells with Lipofectin 2000 (Invitrogen-Thermo Fisher Scientific, Waltham, MA, USA) to suppress the expression of regulatory subunit of calpain. After 36 h of the initial transfection and treatment, cell lysates were collected and analyzed using Western blotting to confirm the expressions of proteins.

### Western blot analysis

Whole cell lysates were prepared and analyzed by Western blotting as described previously [32]. Proteins in cell lysates were separated by precast 8-20% SDS-polyacrylamide gel electrophoresis, and then electrophoretically transferred from the gel onto polyvinylidene difluoride membranes. After blocking, blots were incubated with anti-GRP78, anti-GRP94, anti-phospho-PERK, anti-caspase-12, anti-caspase-7, anti-CHOP, anti-PARP, and anti-β-actin antibodies (Santa Cruz Biotechnology) and anti-eIF2α (Cell Signaling Technology, Danvers, MA, USA) in PBS within 0.1% Tween 20 for 1 h followed by two 15 min washes in PBS with 0.1% Tween 20. The membranes were then incubated with horseradish peroxidase-conjugated secondary antibodies for 60 min. Detection was performed with Western blotting reagent ECL (Amersham-GE Healthcare Life Sciences, Pittsburgh, PA, USA), and chemiluminescence was exposed by the Kodak X-Omat films.

### Statistical analyses

Results are expressed as means ± SEM. For multiple comparisons, results were analyzed by ANOVA followed by Fisher's test. *P*<0.05 was considered statistically significant.

## References

[1] Pfohl-Leszkowicz A, Manderville RA (2007). Ochratoxin A: An overview on toxicity and carcinogenicity in animals and humans. Mol Nutr Food Res.

[2] Pavlovic NM (2013). Balkan endemic nephropathy-current status and future perspectives. Clin Kidney J.

[3] Hald B (1991). Porcine nephropathy in Europe. IARC Sci Publ.

[4] Dwivedi P, Burns RB, Maxwell MH (1984). Ultrastructural study of the liver, kidney in ochratoxicosis A in young broiler chicks. Res Vet Sci.

[5] Ciarcia R, Damiano S, Squillacioti C, Mirabella N, Pagnini U, Florio A, Severino L, Capasso G, Borrelli A, Mancini A, Boffo S, Romano G, Giordano A (2016). Recombinant Mitochondrial Manganese Containing Superoxide Dismutase Protects Against Ochratoxin A-Induced Nephrotoxicity. J Cell Biochem.

[6] Schaaf GJ, Nijmeijer SM, Maas RF, Roestenberg P, de Groene EM, Fink-Gremmels J (2002). The role of oxidative stress in the ochratoxin A-mediated toxicity in proximal tubular cells. Biochim Biophys Acta.

[7] Li J, Yin S, Dong Y, Fan L, Hu H (2011). p53 activation inhibits ochratoxin A-induced apoptosis in monkey and human kidney epithelial cells via suppression of JNK activation. Biochemical and biophysical research communications.

[8] Petrik J, Zanic-Grubisic T, Barisic K, Pepeljnjak S, Radic B, Ferencic Z, Cepelak I (2003). Apoptosis and oxidative stress induced by ochratoxin A in rat kidney. Arch Toxicol.

[9] Taniguchi M, Yoshida H (2015). Endoplasmic reticulum stress in kidney function and disease. Curr Opin Nephrol Hypertens.

[10] Boussabbeh M, Ben Salem I, Prola A, Guilbert A, Bacha H, Abid-Essefi S, Lemaire C (2015). Patulin induces apoptosis through ROS-mediated endoplasmic reticulum stress pathway. Toxicol Sci.

[11] Vrabcheva T, Petkova-Bocharova T, Grosso F, Nikolov I, Chernozemsky IN, Castegnaro M, Dragacci S (2004). Analysis of ochratoxin A in foods consumed by inhabitants from an area with balkan endemic nephropathy: a 1 month follow-up study. J Agric Food Chem.

[12] Castegnaro M, Canadas D, Vrabcheva T, Petkova-Bocharova T, Chernozemsky IN, Pfohl-Leszkowicz A (2006). Balkan endemic nephropathy: role of ochratoxins A through biomarkers. Mol Nutr Food Res.

[13] Joint FAO/WHO (2001). Expert Committee on Food Additives. Meeting (56th: 2001: Geneva Switzerland), World Health Organization., International Program on Chemical Safety. and Food and Agriculture Organization of the United Nations. Safety evaluation of certain mycotoxins in food.

[14] Petkova-Bocharova T, Chernozemsky IN, Castegnaro M (1988). Ochratoxin A in human blood in relation to Balkan endemic nephropathy and urinary system tumours in Bulgaria. Food Addit Contam.

[15] Steyn PS, Vleggaar R (1986). International Union of Pure and Applied Chemistry. Mycotoxins and phycotoxins: a collection of invited papers.

[16] Stoev SD, Stoeva JK, Anguelov G, Hald B, Creppy EE, Radic B (1998). Haematological, biochemical and toxicological investigations in spontaneous cases with different frequency of porcine nephropathy in Bulgaria. Zentralbl Veterinarmed A.

[17] Stoev SD, Hald B, Mantle PG (1998). Porcine nephropathy in Bulgaria: a progressive syndrome of complex or uncertain (mycotoxin) aetiology. Vet Rec.

[18] Maaroufi K, Achour A, Hammami M, el May M, Betbeder AM, Ellouz F, Creppy EE, Bacha H (1995). Ochratoxin A in human blood in relation to nephropathy in Tunisia. Hum Exp Toxicol.

[19] Krogh P, Axelsen NH, Elling F, Gyrd-Hansen N, Hald B, Hyldgaard-Jensen J, Larsen AE, Madsen A, Mortensen HP, Moller T, Petersen OK, Ravnskov U, Rostgaard M (1974). Experimental porcine nephropathy. Changes of renal function and structure induced by ochratoxin A-contaminated feed. Acta Pathol Microbiol Scand Suppl.

[20] Gekle M, Silbernagl S (1993). Mechanism of ochratoxin A-induced reduction of glomerular filtration rate in rats. J Pharmacol Exp Ther.

[21] Gekle M, Silbernagl S (1996). Renal toxicodynamics of ochratoxin A: a pathophysiological approach. Kidney Blood Press Res.

[22] Gill PS, Wilcox CS (2006). NADPH oxidases in the kidney. Antioxidants & redox signaling.

[23] Liang R, Shen XL, Zhang B, Li Y, Xu W, Zhao C, Luo Y, Huang K (2015). Apoptosis signal-regulating kinase 1 promotes Ochratoxin A-induced renal cytotoxicity. Sci Rep.

[24] Schwerdt G, Holzinger H, Konigs M, Humpf HU, Gekle M (2009). Effect of ochratoxin A on cell survival and collagen homeostasis in human mesangial cells in primary culture. Food Chem Toxicol.

[25] Xia K, He X, Dai Q, Cheng WH, Qi X, Guo M, Luo Y, Huang K, Zhao C, Xu W (2014). Discovery of systematic responses and potential biomarkers induced by ochratoxin A using metabolomics. Food Addit Contam Part A Chem Anal Control Expo Risk Assess.

[26] Peiro C, Romacho T, Azcutia V, Villalobos L, Fernandez E, Bolanos JP, Moncada S, Sanchez-Ferrer CF (2016). Inflammation, glucose, and vascular cell damage: the role of the pentose phosphate pathway. Cardiovasc Diabetol.

[27] Wang D, Chen Y, Chabrashvili T, Aslam S, Borrego Conde LJ, Umans JG, Wilcox CS (2003). Role of oxidative stress in endothelial dysfunction and enhanced responses to angiotensin II of afferent arterioles from rabbits infused with angiotensin II. Journal of the American Society of Nephrology: JASN.

[28] Marin M, Giner RM, Rios JL, Recio Mdel C (2013). Protective effect of apocynin in a mouse model of chemically-induced colitis. Planta Med.

[29] Ozbek O, Altintas R, Polat A, Vardi N, Parlakpinar H, Sagir M, Duran ZR, Yildiz A (2015). The protective effect of apocynin on testicular ischemia-reperfusion injury. J Urol.

[30] Kilic T, Parlakpinar H, Taslidere E, Yildiz S, Polat A, Vardi N, Colak C, Ermis H (2015). Protective and therapeutic effect of apocynin on bleomycin-induced lung fibrosis in rats. Inflammation.

[31] Ciarcia R, Damiano S, Florio A, Spagnuolo M, Zacchia E, Squillacioti C, Mirabella N, Florio S, Pagnini U, Garofano T, Polito MS, Capasso G, Giordano A (2015). The Protective Effect of Apocynin on Cyclosporine A-Induced Hypertension and Nephrotoxicity in Rats. J Cell Biochem.

[32] Fan R, Shan X, Qian H, Song C, Wu G, Chen Y, Miao Y, Cha W (2012). Protective effect of apocynin in an established alcoholic steatohepatitis rat model. Immunopharmacol Immunotoxicol.

[33] Ahmad A, Mondello S, Di Paola R, Mazzon E, Esposito E, Catania MA, Italiano D, Mondello P, Aloisi C, Cuzzocrea S (2012). Protective effect of apocynin, a NADPH-oxidase inhibitor, against contrast-induced nephropathy in the diabetic rats: a comparison with n-acetylcysteine. Eur J Pharmacol.

[34] Chen F, Li Q, Zhang Z, Lin P, Lei L, Wang A, Jin Y (2015). Endoplasmic Reticulum Stress Cooperates in Zearalenone-Induced Cell Death of RAW 264.7 Macrophages. Int J Mol Sci.

[35] Liu X, Schnellmann RG (2003). Calpain mediates progressive plasma membrane permeability and proteolysis of cytoskeleton-associated paxillin, talin, and vinculin during renal cell death. J Pharmacol Exp Ther.

[36] Chatterjee PK, Todorovic Z, Sivarajah A, Mota-Filipe H, Brown PA, Stewart KN, Mazzon E, Cuzzocrea S, Thiemermann C (2005). Inhibitors of calpain activation (PD150606 and E-64) and renal ischemia-reperfusion injury. Biochem Pharmacol.

[37] Muruganandan S, Cribb AE (2006). Calpain-induced endoplasmic reticulum stress and cell death following cytotoxic damage to renal cells. Toxicol Sci.

[38] Sanvicens N, Gomez-Vicente V, Masip I, Messeguer A, Cotter TG (2004). Oxidative stress-induced apoptosis in retinal photoreceptor cells is mediated by calpains and caspases and blocked by the oxygen radical scavenger CR-6. J Biol Chem.

[39] Gorlach A, Bertram K, Hudecova S, Krizanova O (2015). Calcium and ROS: A mutual interplay. Redox Biol.

[40] Sheu ML, Ho FM, Chao KF, Kuo ML, Liu SH (2004). Activation of phosphoinositide 3-kinase in response to high glucose leads to regulation of reactive oxygen species-related nuclear factor-kappaB activation and cyclooxygenase-2 expression in mesangial cells. Mol Pharmacol.

[41] Vaquero EC, Edderkaoui M, Pandol SJ, Gukovsky I, Gukovskaya AS (2004). Reactive oxygen species produced by NAD(P)H oxidase inhibit apoptosis in pancreatic cancer cells. J Biol Chem.

[42] Sheu ML, Ho FM, Yang RS, Chao KF, Lin WW, Lin-Shiau SY, Liu SH (2005). High glucose induces human endothelial cell apoptosis through a phosphoinositide 3-kinase-regulated cyclooxygenase-2 pathway. Arterioscler Thromb Vasc Biol.

